# Operation Melilli, forensic humanitarian medico legal activities on the largest known Mediterranean disaster: the shipwreck of April 18th, 2015

**DOI:** 10.1007/s00414-026-03886-y

**Published:** 2026-08-07

**Authors:** Cristina Cattaneo, Debora Mazzarelli, Annalisa Cappella, Davide Porta, Pasquale Poppa, Vera Merelli, Francesca Magli, Alberto Amadasi, Lara Olivieri, Giulia Caccia, Andrea Palamenghi, Marco Cummaudo, Annalisa D’Apuzzo, Egidio Ibba, Davide Damante, Nicola De Felice, Giuseppe Romano, Luca Cari, Luca Maria Sconfienza, Grazia Pozzi, Carmelo Messina, Nunziata Giuseppa Elisabetta Barbera, Antonina Argo, Stefania Zerbo, Giuseppe Lo Re, Alessio Asmundo, Daniela Sapienza, Roberto Giuffrida, Denise Albani, Elena Pilli, Gianpietro Lago, Alberto Marino, Diego Denari, Luca Tajana, Barbara Bertoglio, Pierangela Grignani, Carlo Previderé, Carlo Robino, Andrea Verzeletti, Chiara Rossetti, Lorenzo Franceschetti, Luca Salvaderi, Adriano Tagliabracci, Mauro Pesaresi, Antonio De Donno, Valeria Santoro, Rossana Cecchi, Antonio Banchini, Rosa Maria Gaudio, Letizia Alfieri, Susi Pelotti, Daniela Roberta Schillaci, Lucia Tattoli, Giancarlo Di Vella, Richard L. Jantz, Douglas H. Ubelaker, Rossella Di Liberto, Jose-Pablo Baraybar, Vittorio Piscitelli, Danilo De Angelis

**Affiliations:** 1 LABANOF, Laboratorio di Antropologia e Odontologia Forense, Sezione di Medicina Legale, Dipartimento di Scienze Biomediche per la Salute, Università degli Studi di Milano, Via L. Mangiagalli 37, 20133 Milan, Italy; 2 Dipartimento di Scienze Biomediche per la Salute, Università degli Studi di Milano, Milan, Italy; 3 Impresub Diving Marine Contractor, Trento, Italy; 4 Comando Marittimo Sicilia della Marina Militare Italiana, Augusta, Italy; 5 Corpo Nazionale Vigili del Fuoco, Rome, Italy; 6 IRCCS Istituto Ortopedico Galeazzi, Milan, Italy; 7 Dipartimento di Scienze Mediche, Chirurgiche e Tecnologie Avanzate, Università degli Studi di Catania, Catania, Italy; 8 Dipartimento di Scienze per la Promozione della Salute e Materno Infantile “G. D’Alessandro”, Università degli Studi di Palermo, Palermo, Italy; 9 Dipartimento di Biopatologia e Biotecnologie Mediche, Università degli Studi di Palermo, Palermo, Italy; 10 Dipartimento di Scienze Biomediche, Odontoiatriche e delle Immagini Morfologiche e Funzionali, Sezione di Medicina Legale, Università degli Studi di Messina, Messina, Italy; 11 Laboratorio di Genetica Forense, Gabinetto Regionale Polizia Scientifica, Milan, Italy; 12 Laboratorio di Antropologia Molecolare Forense, Dipartimento di Biologia, Università degli Studi di Firenze, Via del Proconsolo 12, Florence, 50122 Italy; 13 Raggruppamento Carabinieri Investigazioni Scientifiche, Parma, Italy; 14 Laboratorio di Genetica Forense, Dipartimento di Sanità Pubblica, Medicina Sperimentale e Forense, Università di Pavia, Pavia, Italy; 15 Dipartimento di Scienze della Salute Pubblica e Pediatriche, Università degli Studi di Torino, Turin, Italy; 16 Dipartimento di Specialità Medico- Chirurgiche, Scienze Radiologiche e Sanità Pubblica, Unità di Medicina Forense, Università degli Studi di Brescia, Brescia, Italy; 17 Sezione di Genetica Forense, Laboratorio Eurofins Genoma Group, Milan, Italy; 18 Dipartimento Scienze Biomediche e Sanità Pubblica, Sezione di Medicina Legale, Università Politecnica delle Marche, Ancona, Italy; 19 Dipartimento Interdisciplinare di Medicina, Sezione di Medicina Legale, Università degli Studi di Bari, Bari, Italy; 20 Dipartimento di Medicina e Chirurgia, Sezione di Medicina Legale, Università di Parma, Parma, Italy; 21 Dipartimento di Morfologia, Chirurgia e Medicina Sperimentale, Università degli Studi di Ferrara, Ferrara, Italy; 22 Dipartimento di Scienze Mediche e Chirurgiche, Unità di Medicina Legale, Università di Bologna, Bologna, Italy; 23 Dipartimento di Medicina e Chirurgia, Università degli Studi di Milano Bicocca, Milan, Italy; 24 S.C. Medicina Legale U, A.O.U. Città della Salute e della Scienza di Torino, Torino, Italy; 25 Department of Anthropology, University of Tennessee, Knoxville, USA; 26 Department of Anthropology, National Museum of Natural History, Smithsonian Institution, Washington, DC USA; 27 Croce Rossa Italiana, Rome, Italy; 28 International Committee of the Red Cross, Paris, France; 29 Ufficio del Commissario Straordinario per le Persone Scomparse, Rome, Italy

**Keywords:** Migrants, Human rights, Europe, Mediterranean Sea, Forensic pathology, Forensic anthropology, Humanitarian forensic action

## Abstract

On April 18th, 2015, a boat carrying about 1,000 migrants sank in the Strait of Sicily, prompting one of the most complex recovery missions ever conducted in the Mediterranean. Operation Melilli was the first time a European Government authorized the recovery of a sunken migrant ship to identify all victims. The Italian Navy and Fire Brigade recovered the wreck and remains, while forensic pathologists, anthropologists, and radiologists from 13 universities, coordinated by the Commissioner for Missing Persons, conducted the postmortem examinations. From the wreck, 528 bodies and 30,000 commingled remains—mostly of young Sub-Saharan men—were retrieved. Collaboration with the International Committee and Italian Red Cross enabled antemortem data collection for over 300 missing persons. Ten years later, 33 victims have been identified through DNA analysis and other 40 are currently being confirmed as operations are still ongoing at the time of the preparation of his article. Beyond the number of identifications achieved so far, Operation Melilli demonstrated that migrant deaths can be addressed with the same forensic rigor, dignity and traceability expected in any other mass disaster. Its humanitarian value lies not only in the identities already restored and to be restored in the future, but also in the systematic recovery, documentation, sampling of the victims and preservation of their personal belongings, which keep future identifications possible. This operation also prompts broader considerations regarding resource allocation, international cooperation, and ethical responsibility in responding to such events. As such, Operation Melilli stands therefore as enduring evidence of how forensic science can serve humanitarian goals. Our experience also shows that these tasks are feasible and would not require extreme financial resources from the EU but rather more efficient collection and exchanges of ante and postmortem data at least among European countries. In the words of then-Italian Prime Minister Matteo Renzi (2014–2016): “*We are human*,* there are 500*,* 600 people*,* children who died in this manner. I think it is right for ltaly to reveal its centuries of civilization. Let’s go and recover that boat […] I want the entire world to see”*.

## Introduction

Each mass disaster demands a tailored approach that considers the specific nature, scale, and circumstances of the event. Factors such as the cause, the type of the disaster (open vs. closed), the number of victims, the condition of the remains, the environment in which the incident occurred, and the available resources all influence the strategies employed for recovery, identification, and documentation. As a result, forensic teams must adapt their methods to effectively address the unique challenges presented by each situation, ensuring a respectful and scientifically sound response. Among the complicated mass disaster scenarios that forensic experts deal with, migration seems to be the most demanding plight. Political crises, climate change, persecution and warfare force people to flee their home countries and to embark on long and trying journeys that often lead to their death. Fatal mass migrations occur globally but are documented with a higher incidence in some crucial geographical areas, such as the Mediterranean basin, Africa and the Southern U.S borders [1–3]. 2024 was the deadliest year on record for migrants worldwide, and it is estimated that one in four migrant deaths occurred in the central Mediterranean Sea [4]. In fact, the central Mediterranean Sea route is considered the most dangerous and still registers the greatest number of victims, representing 50–60% of the global fatalities. Migrants often attempt to take the central Mediterranean route and cross the strait of Sicily, aboard overcrowded and cramped boats, decrepit seiners and small dinghies departing from North African countries. Unfortunately, these tragic journeys often result in death. Unlike sudden, large-scale catastrophes, these incidents unfold gradually, often across vast geographic areas and extended periods, hence they can be considered a mass disaster diluted in time and space. Despite their less immediate impact, the cumulative effect can be equally devastating, necessitating coordinated forensic and humanitarian responses [5–7]. Given its geographical location, Europe and Italy have witnessed in the last 10 years hundreds of landings and shipwrecks that resulted in mass fatalities. According to the IOM Missing Migrants project, more than 10,000 bodies have been found and over 32,000 people are reported as dead or missing in the Mediterranean region since 2014 [2, 3], but this is obviously just the tip of the iceberg as victims are not retrieved from the bottom of the sea after shipwrecks. Furthermore, the current tragedy of these deaths obscures a less evident one which concerns the toll of unidentified bodies. Tragically, the low numbers of positive identifications of dead migrants are striking: 65% of the recovered bodies in Europe are still unidentified and remain nameless [8]. Despite this, the unresponsiveness of the member states of the European Union in facing this humanitarian crisis continues to contribute to this plight [2].

Since 2013, Italy has committed to the cause of identifying dead migrants. This was possible through the joint efforts of LABANOF (the Forensic Anthropology Laboratory of the University of Milano), the Office of the Commissioner for Missing Persons of the Italian Government, Police, other Agencies of the Ministry of Interiors, several prosecutors’ courts, several Italian Universities, along with NGOs such as the ICRC, the Italian red Cross, Comitato 3 ottobre and the IOM. Following the disaster of October 3rd and 11th 2013 [9], and encompassing in this activity many other disasters [10] efforts were directed not only toward achieving identification but also toward raising awareness and establishing both a protocol and a network to facilitate the identification process (in other words collection of postmortem data from various prosecutors’ offices and antemortem data from the relatives). These 10 years of experience have proven that not only is it mandatory and possible to identify these victims regardless of the difficulties, but that the largest obstacle (in Europe and the Mediterranean) lies in the attitude of European states in not wanting to face these obligations (it would in fact be relatively simple by implementing already existing forensic offices) and not treating these dead and their living families with the same procedures that would be deployed in cases of “domestic” disasters [11–13].

On April 18th, 2015, a fishing boat coming from the Libyan shores and allegedly carrying about 1,000 people, capsized in the strait of Sicily and sank 143 km north from the Libyan coast and 177 km south of the Italian island of Lampedusa (at 76 Nautical Miles-NM- from the Libyan coast, 112 NM from Malta and 131 NM from Lampedusa), with only 28 survivors. This case report describes the only exception to this unresponsive European mindset, which was supported by the Italian government, where, at least in part, and with the *pro bono* work of many academics, a disaster involving a large fishing boat carrying about 1,000 victims, was dealt with and treated in a standard fashion, at least for what concerns the postmortem activities. For the first time the world of academia, the Ministry of Interior, the Italian Navy, the National Fire and Rescue Service, the prosecutor’s office and many other national agencies embraced this tragedy as if it were “their own”. This report therefore aims to present the case of the disaster of April 18th, 2015.

## Initial recovery of the bodies around the sunken boat: the operations of the Italian Navy and Fire Department

In the immediate aftermath of the disaster, 24 bodies were recovered and taken to the island of Malta. The Italian Navy located the shipwreck lying on the seabed at a depth of 375 m, noticing that it was surrounded by dozens of cadavers who presumably had fallen out of the boat, and decided to begin recovering the bodies outside the vessel in July 2015, three months after the shipwreck. All the recovery operations were carried out by the Italian Navy and The National Fire and Rescue Service, under the supervision and in accordance with the public prosecutor’s office of Catania and the police forces *Squadra Mobile di Catania e Polizia Scientifica*,* and ASP Sicilia (the local health authority).* The first operation took place in collaboration with the scuba divers of the Italian Navy (*Gruppo Operativo Subacquei*,* Comsubin)* between July 2015 and January 2016 and concerned the recovery of the non-sequestered (i.e., outside the wreck) bodies. The bodies (*N* = 169) were retrieved from the sea bottom using the arm of a Remotely Operated Vehicle (R.O.V.) due to the prohibitive depth for diving operations, and were placed inside individual body bags [14]. At autopsy, each body was assigned a number according to the Interpol international PM numbering system and preserved inside a mortuary refrigerating unit at -20 °C.

Almost one year later, not without animated discussions within parliament, Matteo Renzi, prime Minister at the time, decided to recover the entire boat. Hence, the second operation started with the recovery of the shipwreck and of the bodies within it. The specialized company Impresub Diving Marine Contractor, based in Trento - Italy, had been commissioned by the Navy for all underwater intervention activities aimed at the recovery of the wreck, thanks to its experience in wrecks recovering in deep waters. In fact, for this operation the methodology previously developed by Impresub for the recovery of the Albanian refugees’ boat Kater Radez I in 1997 was applied [15]. The 2015 project included different phases to relocate and inspect the wreck on site, design and build a dedicated robotic recovery module and the final underwater recovery operation with wreck delivery to the Italian authorities in Sicily.

**Fig. 1:**
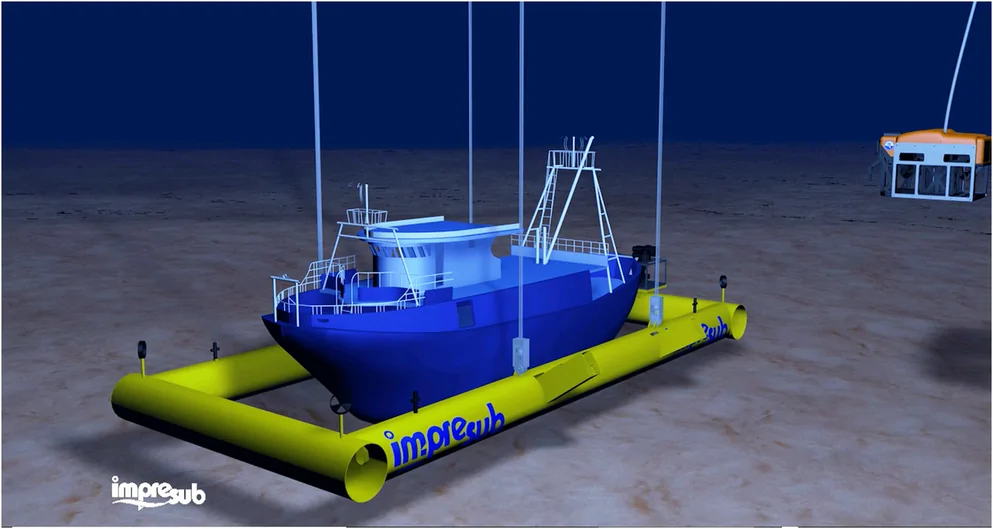
Three-dimensional model of the wreck captured by the robotic recovery module (source: Impresub)

According to the wreck dimensions (27 m long and 7 m wide), a dedicated robotic recovery module had been designed and built. The module, a tubular steel structure approximately 30 m long, was equipped with four hydraulic support hoes, sensors, sonar, cameras and thrusters for rotation and high precision maneuvers. The recovery procedure foresaw the catch of the wreck using the module controlled by the surface and lifting by steel cables using hydraulic strand-jacks from the DP vessel (Fig. 1).

**Fig. 2:**
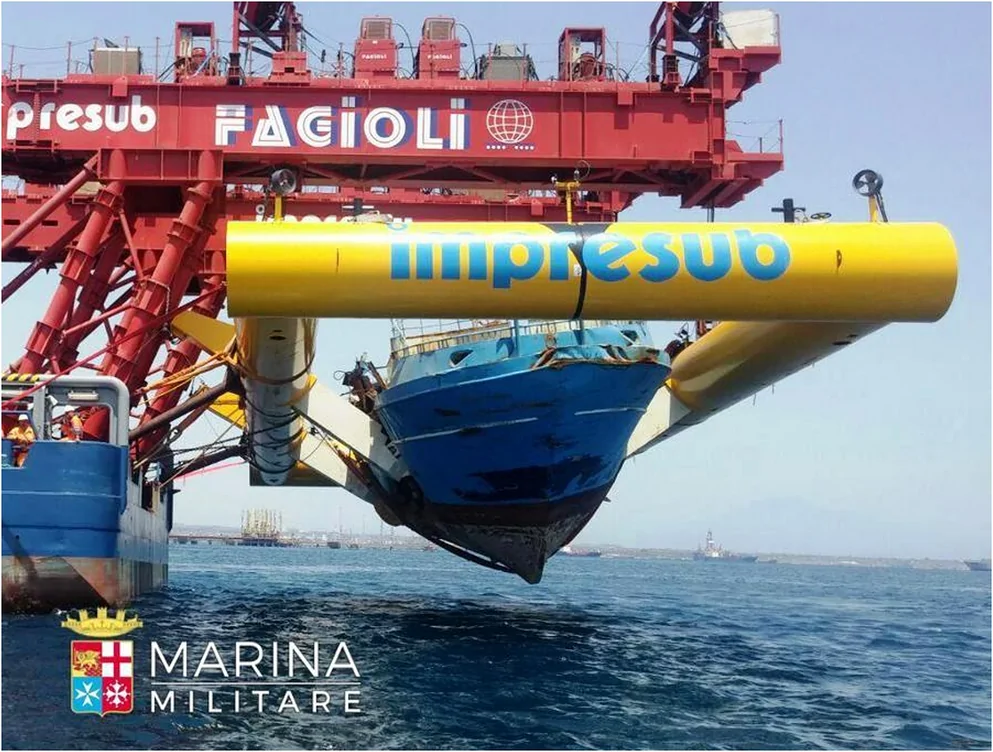
Successful recovery performed in June 2016 by Impresub Diving Marine Contractor for Italian Navy. The Ievoli Ivory DP Vessel hauled the wreck to the Italian shores. (source: Italian Navy)

In this phase, before any intervention on the sunken boat, all the bodies found outside the wreck or immediately on the seabed in its proximity, for a total of 8 corpses, were recovered with the Work Class R.O.V. arms and, once on the surface, handed over to the Navy naval unit on site specially equipped for this purpose. The entire project took many months of processing and failed attempts mainly because of adverse weather conditions. Finally, on June 27th, 2016, the boat was raised inside the module from the seabed and fixed to the lifting systems on board of the *Ievoli Ivory* DP vessel (Fig. 2). All operations were supported by the Italian Navy, present on site with four naval units. Following the transfer in Sicily, the wreck was landed and formally handed over to the Italian authorities at the Melilli NATO base of the Italian Navy on July 30th, 2016.

**Fig. 3:**
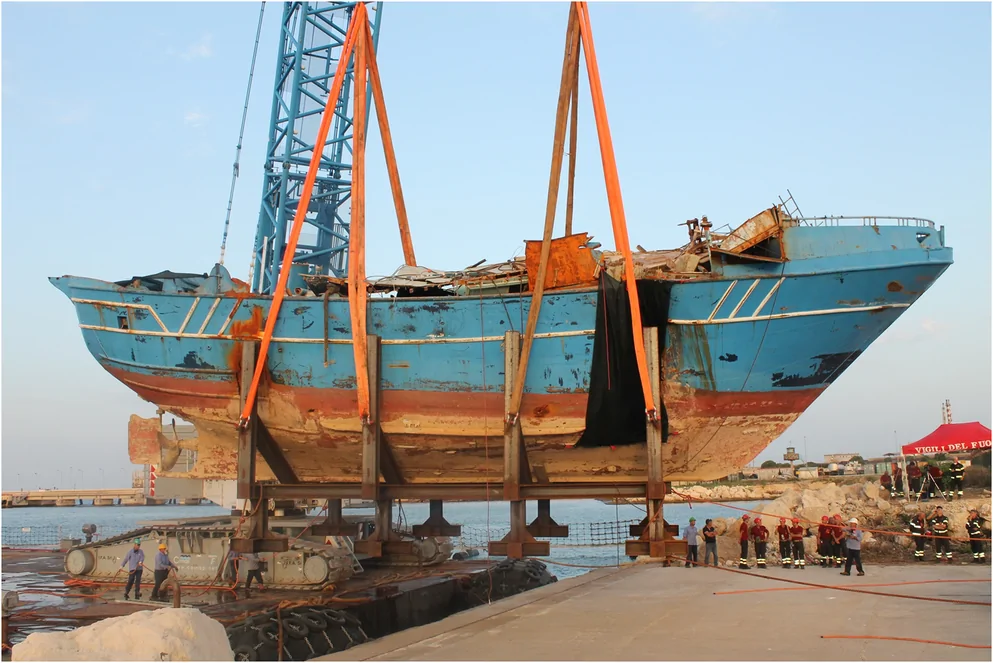
The wreck is moved to land at the Melilli NATO base, where the boat was delivered to the Italian Authorities, Italian Navy and Natural Fire and Rescue Service

Once the wreck was taken to land (Fig. 3), and after medicolegal briefing on how to handle bodies and the Ministry of Health’s green light concerning the lack of specific biological hazards, the firefighters of the National Fire and Rescue Service worked non-stop 24/24 hours for 12 days. Regardless, extra safety procedures were adopted, and the operations were carried out in accordance with CBRN (Chemical, Biological, Radiation and Nuclear) standard operational procedures for medium-low biological risk interventions. All protective devices and decontamination procedures necessary for this type of operation were used. Approximately 1200 decontaminations of firefighters were carried out in total. All firefighters who participated in the operations were involved in preparation briefings and verification de-briefings, also in the presence of psychologists. The Firefighters staff worked following the directions given by the forensic pathologists in order to preserve, as far as possible, the anatomical connection of the remains. The bodies were retrieved, placed in body bags which were given a progressive number that indicated their location in the seiner’s compartments (i.e., main deck, fore and aft peaks, engine room, holds and bilge). In the first days, bodies and remains trapped under the debris on the main deck, the fore and aft peaks were recovered. Then, the wreck was moved under a tensile structure previously prepared with monitored temperature, and the recovery operation of the sequestered bodies inside the boat began. The closed compartments held what looked like an endless expanse of piled up, face down bodies, forming an 80-cm-thick floor made of human bodies and remains. Given the small size of the boat, it can be inferred that it was intended for a crew of 20 people, but the Fire Department estimated that there were at least 5 people/m^2^ in the wreck. The body bags contained human remains with different preservation states and completeness. As such, some body bags contained complete or almost complete bodies and commingled disarticulated bones, whereas some others contained only commingled bones without anatomical connection. Complete and almost complete bodies, and the commingled bones were placed in body bags reporting a number (e.g., body bag n°423). The complete or quasi complete bodies were later given an individual number according to the Interpol international PM (e.g. PM390001) numbering system. Commingled and unassociated parts or bones remained denominated according to the original body bag number.

The number of victims counted in the immediate aftermath of the above-mentioned operations amounted to 528 (136 on deck, stern locker and bow, and 182 in the engine room, bilge and hold, to be added to the 169 (which will turn into 210 found on the seabed by the end of the operations). The bodies were stored in body bags by the workers of the Military Red Cross inside refrigerated tractor-trailer units, awaiting autopsies. At the end of the recovery operations, the wreck was sanitized both internally and externally. In the final intervention of the Italian Navy, the ship *Tremiti* located the last bodies and scattered remains on the bottom of the sea and recovered the last bodies.

## The role of the Commissioner for Missing Persons of the Italian Government (UCPS)

As mentioned previously, Italy had drawn attention to the issue of unknown cadavers since the early 2000s and then to the migrant crisis in the Mediterranean since 2013 [11, 12]. In 2007, the Commissioner for Missing Persons of the Italian Government (UCPS- *Ufficio Commissario straordinario per le Persone Scomparse*) was instated with the aim of facing the dramatic problem of missing persons and unidentified cadavers in Italy. Among its duties, the delegate Commissioner coordinates and monitors the data on missing persons and identification of unknown bodies, and the comparison of antemortem (AM) and postmortem (PM) data. Moreover, the Commissioner promotes the search for the missing, supervising and creating networks between different institutions, universities, non-governmental organizations (NGOs) and the police [10]. Following two previous large shipwrecks which occurred in 2013 in the Mediterranean Sea, the UCPS signed on September 30th, 2014, a memorandum of understanding (MoU) with the University of Milan (especially with its *Laboratorio di Antropologia e Odontologia Forense –* LABANOF) and the Department for Civil Liberty and Immigration to promote action and organize the identification of dead migrants [9, 16]. After the Italian Government decided to recover the bodies of the missing inside the wreck of the April 18th, 2015, disaster, the Government designated the Commissioner for Missing Persons to coordinate and supervise the identification procedures. In July 2015, a new MoU was signed between the Commissioner and the University of Milan, enabling specialized personnel from LABANOF to coordinate the autopsies and the collection of postmortem and, consequently, antemortem data related to this disaster [17]. In 2016, this MoU was implemented by an agreement between the Interior Ministry, the Ministry of Instruction, University and Research and the UCPS Office, that included the technical guidelines for the academic forensic staff of the different universities involved to carry out the examination of the victims.

As concerns the collection of AM data of the missing in African countries, frequently, this task is extremely difficult: one of the main reasons is getting in touch with them to which one must add the issue of safety of relatives and acquaintances which could be endangered in countries where retaliation on relatives of those who leave the country is perpetrated. For the 2013 and 2015 disasters, the UCPS launched a call to all humanitarian organizations and local representatives of foreign communities in Italy, so that relatives and friends of the victims could contact such agencies or the authorities (mainly embassies) and provide antemortem data [9]. Therefore, the role of the UCPS in the resolution of this disaster is paramount because, in addition to the already mentioned activities, this Governmental office acts as an intermediary between forensic investigators in Italy and NGOs working on the antemortem data collection in the countries of origin of the victims. Equally, the role of UCPS is fundamental in the retrieval and use of PM data (autopsy reports, photographs etc.) from this and other disasters from Prosecutors’ offices (which belong to a different Ministry) across the south of Italy, which are otherwise dead-end destinies for such data which may end up dispersed and unavailable in single Prosecutors’ offices. Another important function of UCPS is its role in creating approval of identification reports and sending death certificates directly or through NGOs to the families, which again needs intercession with the Prosecutor’s Office.

## The working facilities at the Melilli naval base

The very first operation had recovered 13 non-sequestered bodies that were then brought and examined at the hospital of Catania where the authorities arranged for an appropriate area in which to perform the autopsies. Following the recovery of other bodies around the wreck, the military base of the Italian Navy in Melilli, Sicily, of the *Comando Marittimo Sicilia* was chosen as the best possible facility. Here, in a building nearby, the Navy also had sleeping barracks and a canteen for workers of the Melilli operation. Furthermore, a camp for the numerous tents of the Fire Brigade, Civil Defence, Italian Red Cross and its military corps was set up. Throughout the entire period required to complete Operation Melilli, the naval base provided room and board (if required) for an average of 150 people per day, including all related services.

**Fig. 4:**
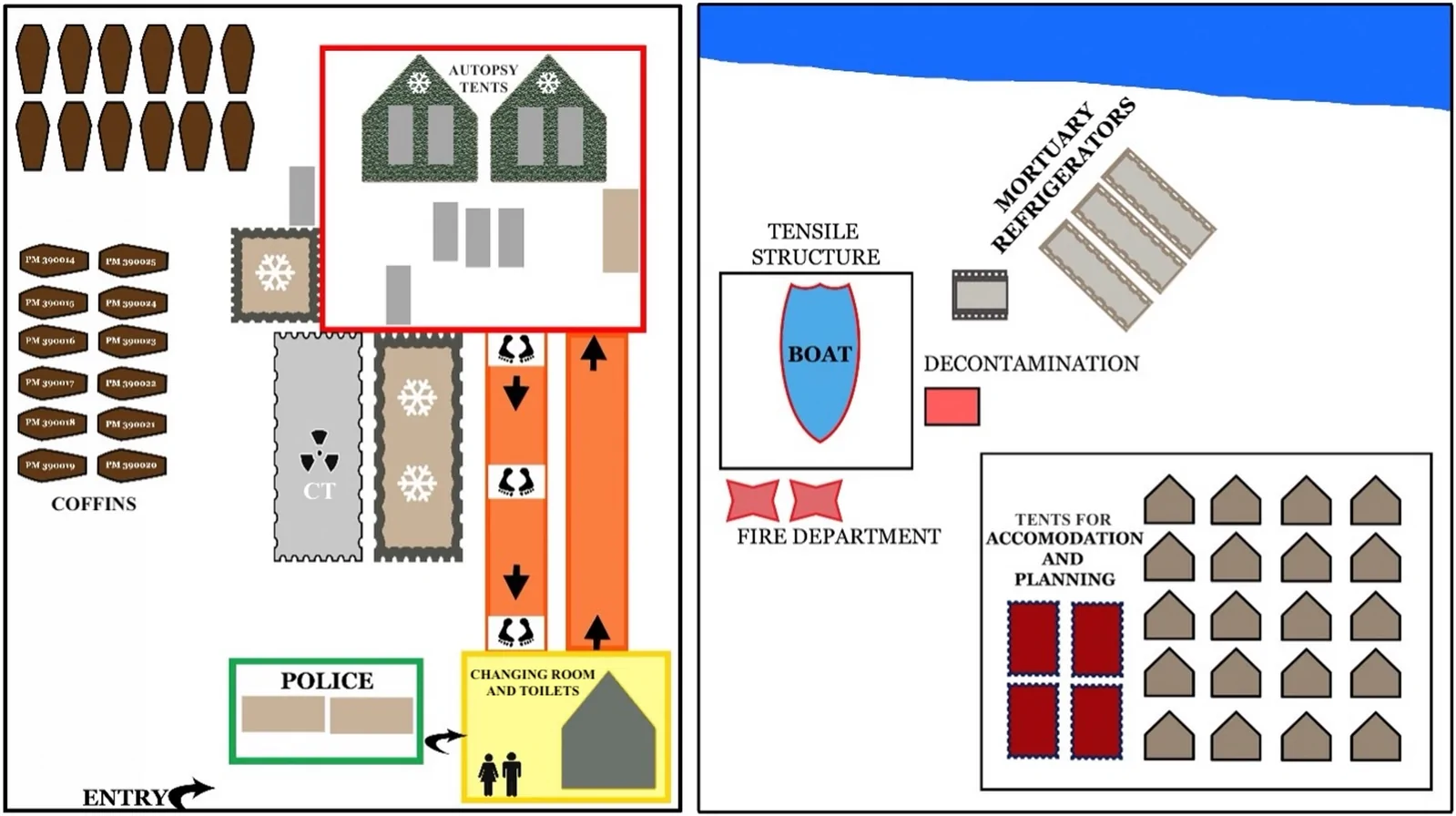
Extemporary morgue at the Melilli base. On the left, area **A**: set up for the postmortem analyses in the hangar. On the right, area **B**: outside space including the tent covering the wreck, decontamination, refrigerators and headquarters in tents of the Italian red Cross, the Civil Protection and the Fire Department

An extemporary morgue in a hangar previously dedicated to the reparation of the engines of ships, was organized in the following manner (Fig. 4).

Area A (Fig. 4, left) was composed of a hangar with a one-way path, in order to avoid cross-contamination of both remains and workers, and included:

- A green area for workers who were not involved in the autopsies, such as police officers of the *Squadra Mobile* who had to register and report all activities, to identify documents and possible signs of foul play to the Prosecutor’s Office.
- A yellow area for the changing room and toilets.
- A red area for medicolegal and anthropological activity on the bodies: along the path between the yellow and red areas, basins were made available for disinfection of PPE (Personal Protective Equipment) worn during the autopsies. The red area was the crucial point where autopsies were performed, and PM data gathered. For this, two tents with water and monitored temperature were mounted, each containing two autopsy tables. In front of the tents was a broad space set up for moving the body bags that were entering and exiting the tents. Right outside the red area there were refrigerator trucks containing the body bags, a mortuary refrigerator for the samples collected during the autopsies and a mobile CT scan.
- Finally, there was an area for coffins containing the bodies and human remains that had been already examined. The setup and non-stop function of the morgue was possible thanks to the collaboration of the Military Red Cross and the staff of the *Comando Marittimo Sicilia*, the Naval Medical Center of the Maritime Command Sicily, the *Brigata S. Marco*, and ASP of Siracusa, i.e. the local and national Health Authority.

Area B (Fig. 4, right) was an open space outside next to the seaside and divided in several sections for different activities. A section was for the Fire Department and Military Cross staff used as accommodation and for planning the activities. Closer to the seaside was the 10 m high tensile structure that protected the wreck once it had been brought to the naval base of Melilli. Next to it was a parking lot for the mortuary refrigerators. Finally, a section was arranged for the decontamination of the workers.

Through this set up, each cadaver (or human remains) was recovered from the boat (or its surroundings), put in a labelled body bag, then in turn thawed and subjected to autopsy in one of the tents during which samples were taken for various analyses, as described in next section. The bodies were then closed into a body bag and into a coffin bearing the same code number of the body. Coffins were stacked in an area adjacent to the two tents and then distributed to various cemeteries across Sicily and Calabria once appropriate authorizations had been released by the prosecutor’s Office and the Prefecture (respectively Ministry of Justice and Interiors) and death certification issued. Furthemore, the following activities carried out by the personnel of the Comando Marittimo Sicilia of the Italian Navy should be stressed, particularly regarding their coordination with all the Prefectures of Sicily and Calabria’s cities for the distribution, transport and burial of 528 bodies in various municipal cemeteries. 

## Postmortem examinations of cadavers and remains and data collection

**Fig. 5:**
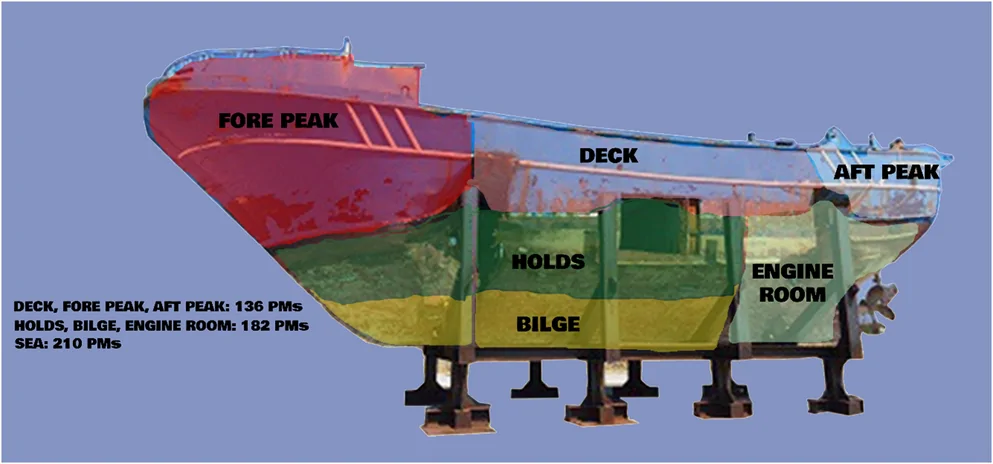
Compartments of the boat, including the related numbers of PM individuals recovered from the sequestered and non-sequestered environments of the boat

It was decided to divide all remains and personal belongings based on their location relative to the wreck (i.e., sea, deck, holds, engine room and bilge) and three main systems of the boat were outlined (Fig. 5) for further reassociation. In the following paragraphs, we will refer to single bodies when in the presence of a complete or quasi complete cadaver representing one person (to which an Interpol code was given, e.g., PM 390001) and to human remains in the presence of commingled bones.

- System A: the sea bottom and in the proximities of the wreck. Where 210 bodies with several human remains were recovered.
- System B: the non-sequestered or partially sequestered environments of the main deck, fore and aft peaks where 136 bodies were recovered together with several unattributed human remains. It is highly likely that commingling occurred between A and B.
- System C: holds, engine room and bilge (apparently- only initially however- sequestered environments) where 182 bodies along with commingled remains were recovered.

Postmortem examinations were carried out over the course of seven missions, spanning a total of 75 days. To this purpose, a task force of 81 forensic practitioners coordinated by the University of Milan was created. The University of Palermo, University of Messina, University of Catania were the first to be recruited and for the longest time, bringing also the expertise of their radiology unit and the mobile CT scan, followed by University of Bari, University *Politecnica delle* Marche, University of Parma, University of Ferrara, University of Bologna, University of Brescia, University of Milano-Bicocca, University of Torino, University of Pavia. Each university deployed forensic pathologists and other forensic scientists on a voluntary basis. The MoU signed in 2016 by the UCPS and University of Milan contained also the guidelines for the medicolegal activities that were to be performed [18]. The human remains presented different stages of decomposition and preservation, including complete cadavers, which as mentioned were given a PM number, partially or fully skeletonized parts, which are denominated according to the body bag number and a letter (e.g., 346-A), and single bones and crania which were given the denomination bag number and a sequential number (e.g., 346-1) [14]. Sometimes the body bags contained one subject or sets of individuals, or even huge numbers of disarticulated and commingled skeletal remains, complicating the medico legal and anthropological activities. Occasionally, only strewn clothes that did not contain human remains were recovered.

**Fig. 6:**
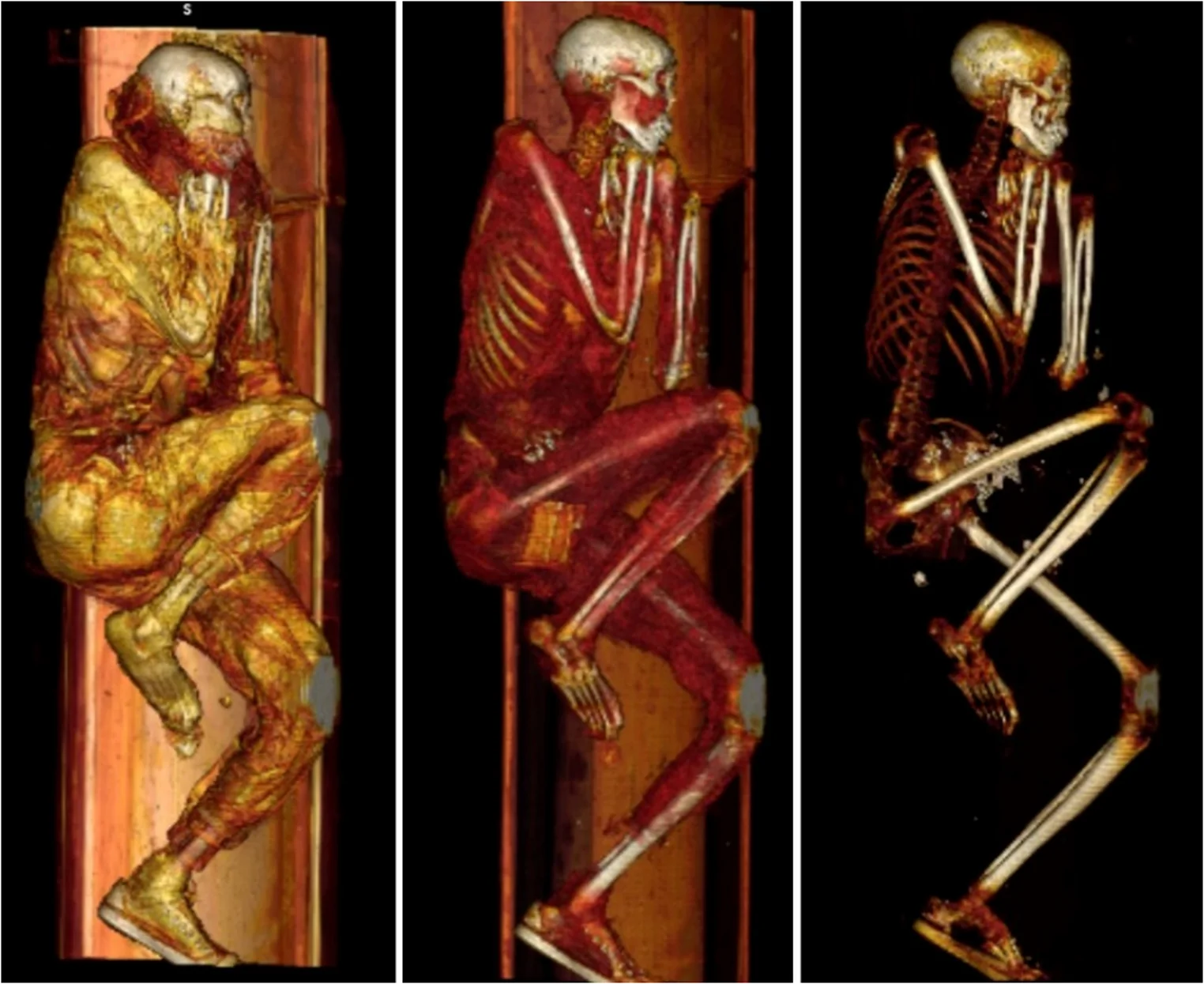
Volume rendering from CT-scan of a PM individual. The virtual removal of the layers allowed to visualize and recover personal items hidden in the clothes. In the middle, a wallet (yellow square on the right thigh) was found in the right posterior pocket

A significant sample of body bags (*N* = 149) was subjected prior to autopsy by the staff of the Radiology Department of the University of Palermo (Fig. 6) to the portable CT scan (BrightSpeed 8 slices CT scanner - GE Healthcare, Milwaukee, WI), which was provided by General Electric (GE) and was available however only for the first two weeks. Its use proved extremely valuable, particularly in the initial stages of the operation, as it helped identify areas where victims had hidden personal belongings and documents — sometimes even sewn into their clothing.

From a medicolegal perspective, this shipwreck can be considered as a pilot project for the PM data collection of such an enormous mass disaster. Given the complicated situation and the fact that forensic practitioners were facing an unprecedented scenario, guidelines that would enhance the current international directives in case of similar incidents were created as well as different ad-hoc recording forms for PM data collection, following the Interpol-DVI guidelines [19].

The type of remains could be classified into (a) complete or almost complete bodies (where at least more than half of the body was articulated, usually with soft tissue and viscera); (b) articulated portions of bodies (e.g., single limbs); (c) commingled bones and teeth. Each had its own recording form as described below.

- a) Complete or almost complete bodies (PM). As per standard autopsy procedures, the bodies underwent external and internal examination with a close inspection of clothing which included investigating seams and internal linings given what had been verified by the initial CT scans. Bone and dental samples for sex (when sex had not been obvious at the external exam through assessment of the external genitalia of the well-preserved bodies or even of the decomposed bodies where remnants of genital organs were still identifiable), age-at-death, population affinity and stature estimations were examined and/or collected. Other samples that were collected included epidermal gloves for fingerprint analyses; skin portions that presented tattoos; head and pubic hair; and sections of femur or tibia for DNA typing. At the end of the examinations, the complete or partially complete cadavers, identified by sequential postmortem codes (i.e., PM390001, PM390002 and so on) were placed in individual coffins to be buried in cemeteries across Southern Italy.

**Fig. 7:**
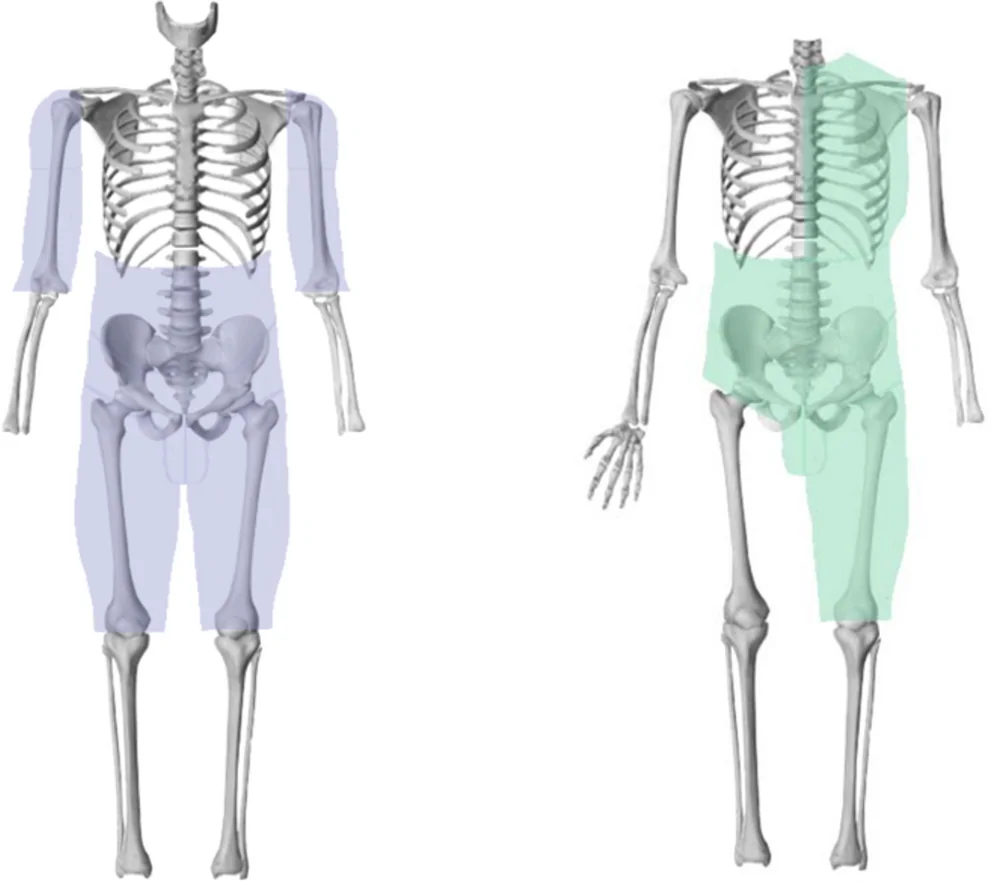
Examples of PM bodies with different states of preservation. Light blue indicates putrefied soft tissues. Green indicates the body parts with adipocere. The other parts were fully or almost skeletonized

For these bodies a postmortem form was designed which was adapted and simplified from the Interpol DVI and from the ICRC PM forms (as not all forensic practitioners were familiar with the most diffuse official PM and AM forms). This form reports the main information required by most PM standard forms: postmortem changes and the state of preservation, e.g., putrefaction, saponification, skeletonization (Fig. 7), the completeness of the body (an inventory of the portions represented and of the skeletal remains), preliminary sex, age-at-death, stature, population affinity, personal features, evidence of trauma or pathology, clothing and personal belongings. The forms also report DNA, anthropological and odontological sampling. All samples taken are stored at LABANOF. A total of 528 PM bodies were counted.

- b) Articulated remains: these were not complete enough to be considered as a single individual to be given a PM code and they included isolated portions of thorax, hands, feet, parts of superior and inferior limbs not associated to a main body. These were not buried but were collected, documented and taken to Milano. An appropriate form was created for these remains for inventory and general preliminary biological profile, depending on portions available.

A total of 205 articulated parts were counted and brought to Milano for anthropological or genetic analyses.

- c) Commingled remains (single bones and teeth): this category includes isolated bones including teeth, crania and mandibles. On site these remains were only assessed preliminary (adult versus subadult, male or female where possible), counted and brought to Milano for anthropological and genetic analyses. For these an appropriate form was prepared.

A total of 320 crania, 275 mandibles and over 30,000 postcranial bones and isolated teeth were counted. As for the articulated remains, all commingled remains were brought to Milano for anthropological or genetic analyses.

All personal belongings associated with the cadavers or found scattered inside the wreck were collected.

## After Melilli: laboratory activities

After Melilli, anthropological and genetic analyses began on the over 2000 samples taken from the bodies and on the 320 crania and the postcranial commingled remains. Samples with soft tissues (e.g., pubic symphyses from PM bodies) and destined to anthropological or odontological analyses were macerated and/or cleaned under fresh water. When dry, the bones from PM bodies were labeled with Indian ink, reporting the PM number. Finally, the specimens of PM bodies sampled for DNA were not processed but stored at -20 °C. Articulated parts and commingled bones followed the same preparation procedure, but they were labeled according to labelling during recovery procedures.

- Analyses on samples taken from PM.

The epidermal gloves of 42 PM individuals were collected and stored at -20 °C. After carefully cleaning the papillary ridges with distilled water, the fingerprints were taken [20]. When the ridges were poorly preserved, the prints were taken from the internal surface of the epidermal glove. Forty-one out of 42 subjects returned at least one useful fingerprint. The fingerprints collected were sent to the Forensic Unit of the Italian Police for match analyses, comparing the postmortem data with the fingerprints in the *Sistema automatizzato di identificazione delle impronte* (APFIS- Automated Palmprint and Fingerprint Identification System) and the General Secretary Interpol of Lyon databases. At the moment, no positive matches have been found.

For each PM individual, the bone samples taken onsite were analysed to implement the preliminary biological profile produced at the autopsy. Skeletal sex, when not achievable at autopsy, was determined with morphological features of the pubic bones [21] and, when available, of the cranium [22] (as per commingled remains in the section below). Age-at-death estimation followed epiphyseal fusion, dental development, and articular degenerative changes of standard joints, such as pubic symphysis, fourth rib, auricular surface (as per commingled remains in the section below). When well-preserved, stature was estimated onsite by measuring the length of the cadavers. Otherwise, long bones of the upper and lower limbs were measured, and the length was noted to apply regression formulae [23, 24]. Furthermore, pathological signs and personal identifiers were noted.

b. Analyses on articulated parts and on commingled remains.

An inventory with the disarticulated commingled bones and articulated parts was created. An initial inventory accounts for about 30,000 skeletal commingled remains, of which 50% has been processed. Reassociation of commingled remains to single individuals constitutes the most challenging undertaking of this mission, because of the high degree of disarticulation and commingling of the bones. Currently, the sorting focuses on the pair-matching of bilateral long bones and on the manual articulation of temporomandibular, atlanto-occipital, vertebral, hip and alveolodental joints. Data obtained from the anthropological analyses will be input in the Commingled Remains Analytics (CoRA) web application [25], specifically designed for the inventory and analysis of commingled skeletal remains.

The biological profile of both disarticulated bones and articulated parts is based on the available elements. Sex estimation includes morphological and metric analyses [22, 26–28], and population affinity is estimated through morphometric analyses on the disarticulated crania [26, 29, 30]. Craniometric data were acquired with a microscribe digitizer (kindly made available by the University of Tennessee) and the 3Skull software [31], and compared to known reference collections [32–35]. Age-at-death estimation of subadult individuals includes assessment of epiphyseal union [36] and dental development [37–39]. Given the dearth of reports on age-at-death estimation on skeletal remains from Sub-Saharan countries, standardized methods for other populations (South Africa, United States and Europe) were employed. Age-at-death of adult individuals was estimated by dental [40–44] and skeletal methods [45–49]. Moreover, the anthropological study of these remains included the detection and interpretation of bone and dental markers that can provide information on health status habits, occupational activity and rituals [50, 51]. Pathological and traumatic skeletal signs were recorded and investigated to complete the biological profile [52]. Moreover, radiological analyses (RX and CT scans) on crania, mandibles and long bones were performed thanks to the collaboration with the Radiology department of the *Ospedale Galeazzi* [53].

c. Processing of clothes, personal belongings, and documents.

Clothes were washed on site, photographed and described in the forms. Details regarding their features, kind, size and color were collected and the data inserted in a database. All personal belongings and documents were brought to LABANOF. These were all cleaned – except for the most delicate specimens (photographs, documents and pieces of paper) – photographed, described and, when necessary, translated. The items included passports, ID cards, photographs, passport photos, SIM cards, mobile phones and other electronics devices, necklaces and other ornaments, wallets, banknotes, agendas, certificates, letters, food and drink wraps. Finally, a PDF file with all the pictures of personal effects was created to be potentially shown to the relatives during the interviews. Overall, around 1,000 personal belongings were collected, including 54 ID cards, 39 passports, and other documents such as vaccination certificates, electoral cards, driving licence, UNHCR cards. Nearly 200 of these reported some personal information and were sent out to the ICRC, in order to facilitate the search for relatives in the countries of origin.

d. Genetics.

Together with the anthropological and radiological examinations, the genetic profile of each PM individual and all crania was defined through DNA analysis of the remains in order to eventually re-associate the disarticulated bone elements and correctly identify the victims.

**Fig. 8:**
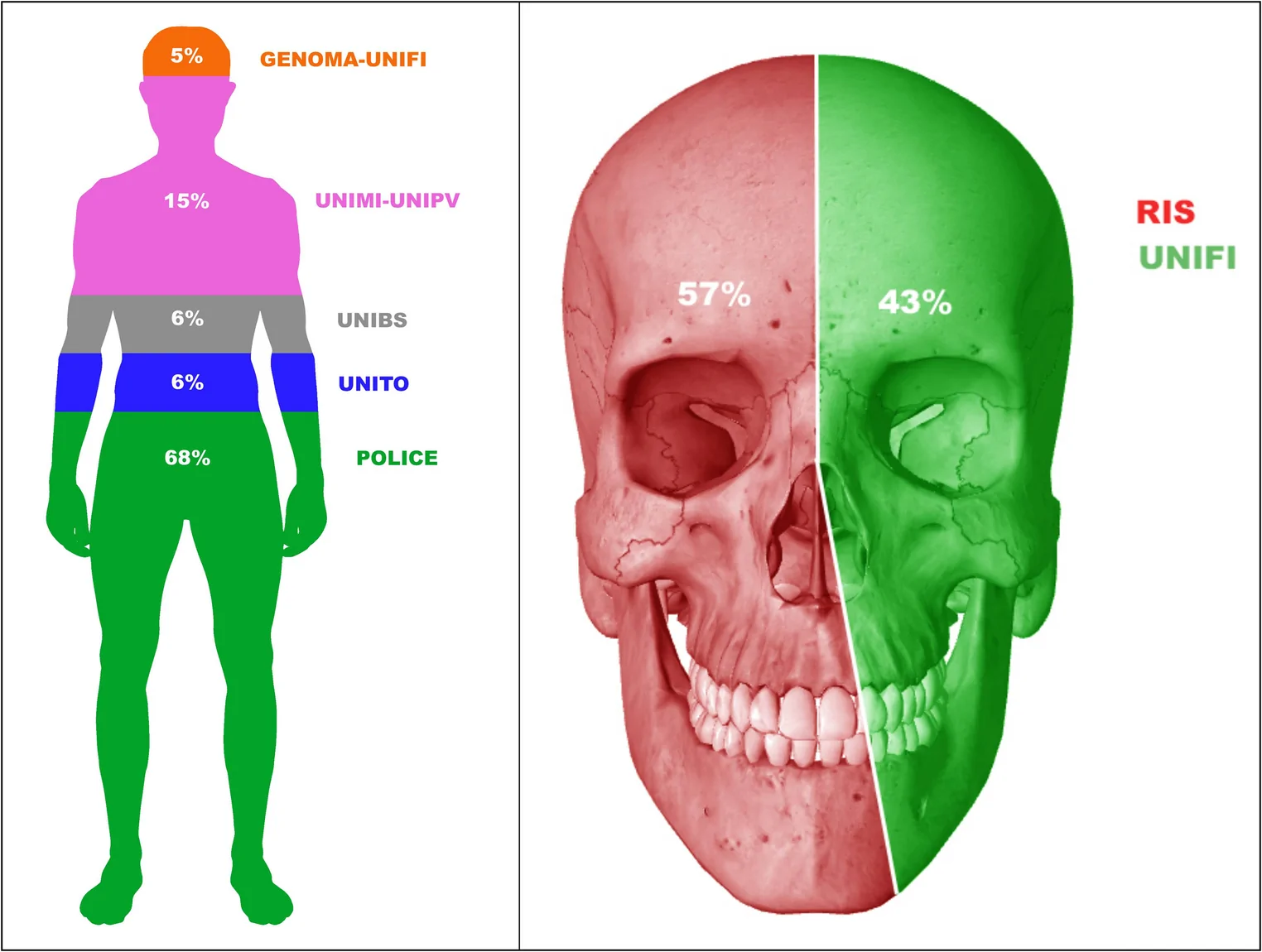
Distribution of the bone sample for genetic analyses to the different Universities and agencies involved in the identification project. On the left, the PM individuals; on the right, the disarticulated crania. The percentages indicate how many samples were appointed to each laboratory

To this purpose, the UCPS launched a call for genetic analyses to be done pro bono which was answered by the Forensic Genetics Laboratories of the Italian Universities of Firenze, Pavia, Torino, Brescia via the Ministry of Instruction University and Research (MIUR), in addition to Forensic Genetics Laboratories affiliated to law enforcement agencies (*Carabinieri* (RIS) and *Polizia Scientifica*) and private molecular genetics laboratories (GENOMA). Samples from the PM bodies and disarticulated crania were taken and distributed to the laboratories (Fig. 8). The petrous pyramid of the temporal bone was selected for sampling [54, 55] in the case of crania whereas femoral samples were taken from the PMs.

Samples were processed in agreement with recommendations [56], using DNA extraction and amplification protocols validated by each participating laboratory. A common set of 21 autosomal STR markers (CODIS + ESS) was selected to achieve genetic profiles at least in duplicate amplifications of a single commercial kit or using multiple kits configurations in order to recover the genetic information from the most challenging samples.

The resulting genetic profiles pooled at Labanof will be compared to the genetic data obtained from the alleged relatives of the missing (AM database) in kinship analysis.

Only at the time of writing of this manuscript were the genetic extractions completed, and comparisons are still ongoing.

e. AM data collection.

Soon after the recovery of the boat, a collaboration with the International Committee of the Red Cross (ICRC) and Italian Red Cross (CRI) for the collection of antemortem (AM) records in the possible countries of origin of the victims led to contacts with several families both in Europe and in the countries of origin such as Mali, Mauritania, Senegal and Ivory Coast. Preliminary information on the provenance were obtained from the identity cards and documents found associated to some bodies. The forensic ICRC unit was deployed in several missions in African countries, such as Mali, Senegal, Mauritania and Ivory Coast, where they interviewed families reporting missing relatives on the boat of April 18th, 2015. For each missing person, an AM form was filled out and sent to UCPS and LABANOF. To date, AM data for more than 300 missing persons reported in this shipwreck has been recovered. For 200 missing persons at least one DNA sample from each family has been recovered and sequenced.

## Results

**Fig. 9:**
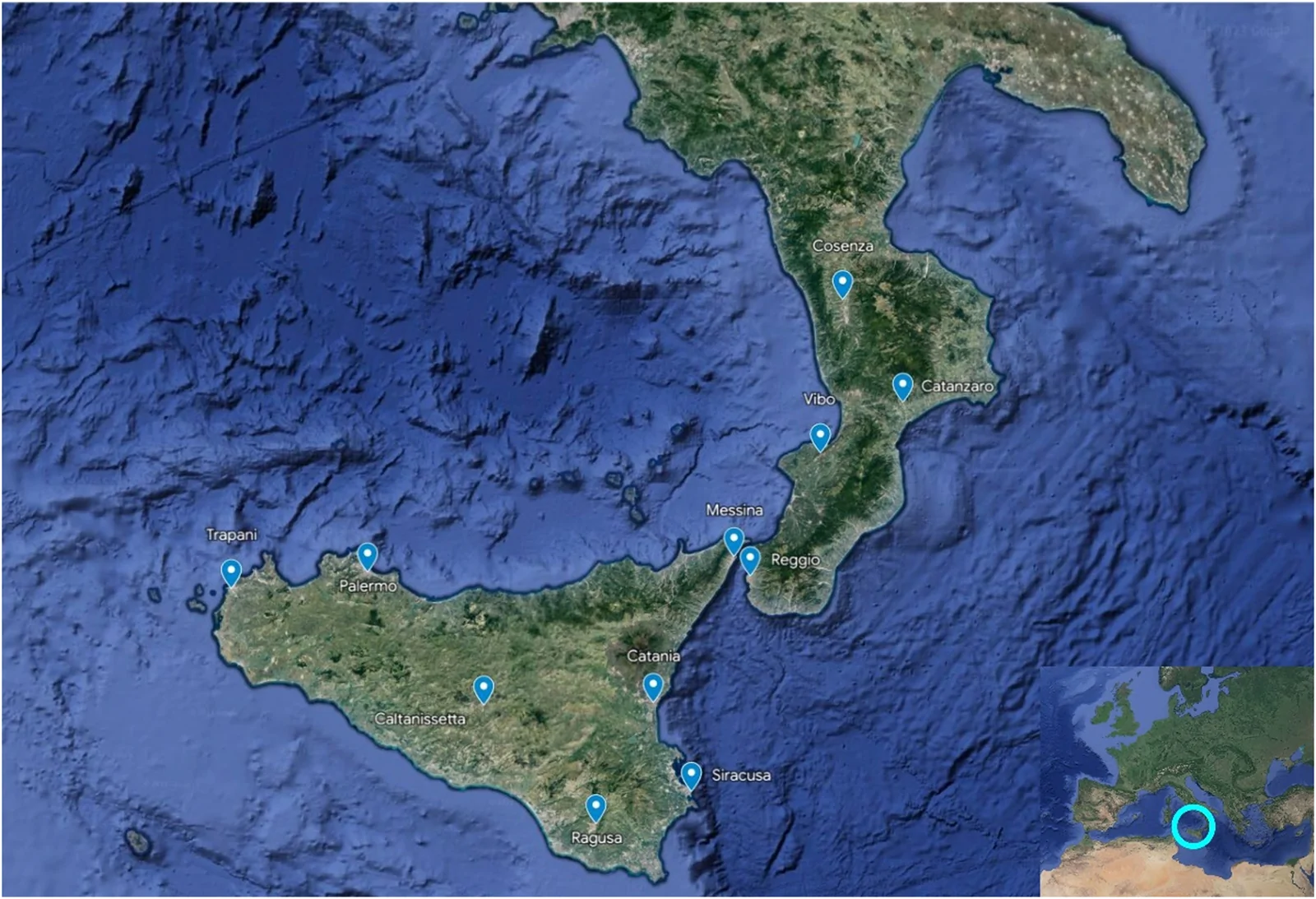
Location of the cemeteries in Southern Italy where the PM individuals were buried

This section summarizes the results of the postmortem examinations and anthropological analyses described above. On survivors’ account, 800 to 1,000 people travelled on the boat, but only 528 complete or partial bodies (i.e., PM individuals) were recovered during Operation Melilli and buried in cemeteries across 11 cities in Southern Italy (Fig. 9). For each PM individual, the exact location of burial is known and can be communicated to the families that are looking for their relatives and loved ones that perished in this disaster. Right after the shipwreck, 24 victims were recovered and taken to Malta, for which Italy does not have autopsy reports or genetic data.

**Fig. 10:**
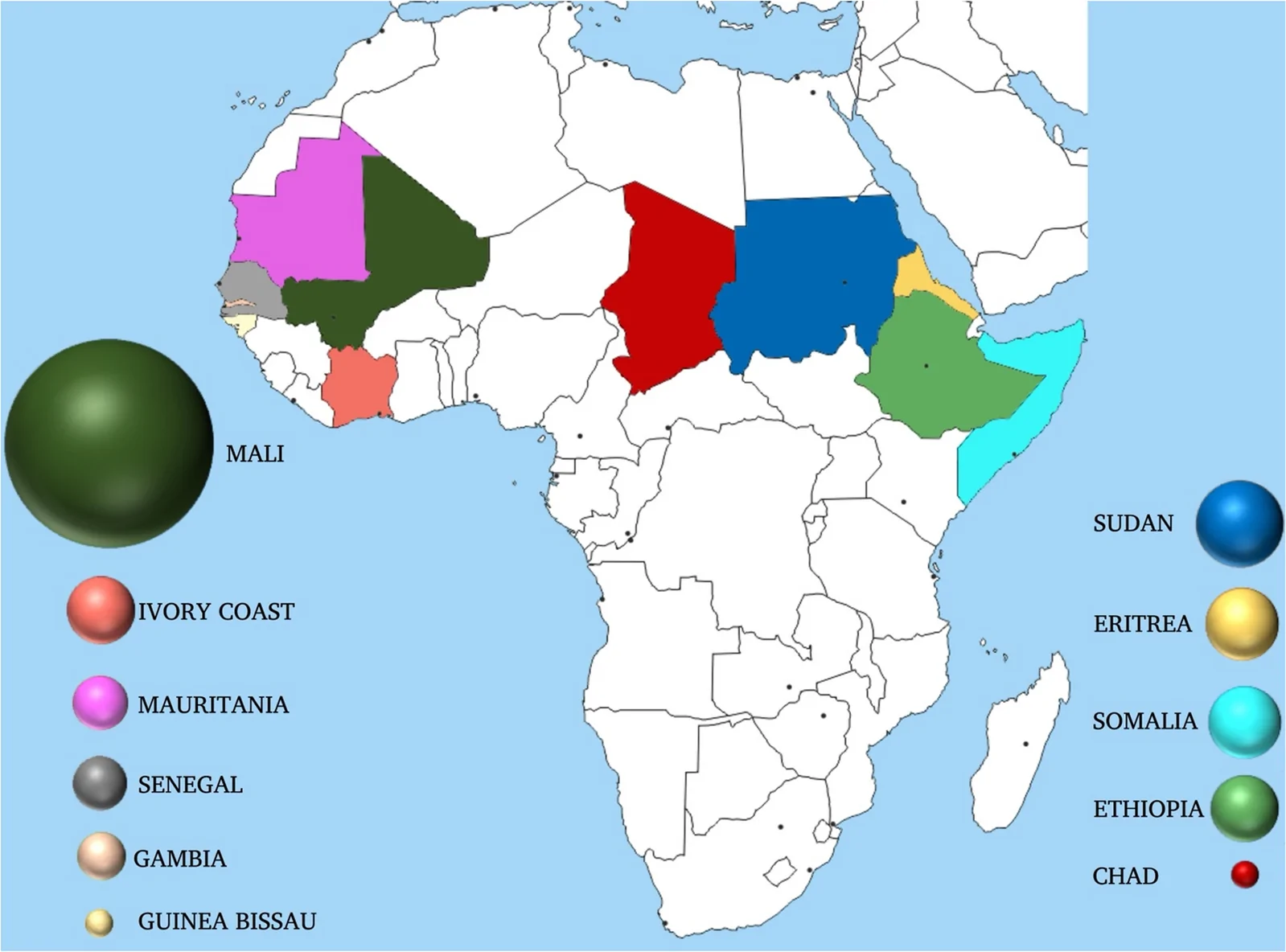
Countries of origin according to the identity cards and documents. The size of the dots indicates the relative quantity of documents from each country

Overall, the medico legal part of Operation Melilli involved 81 forensic experts including forensic pathologists, anthropologists, odontologists and technicians. The Police officers of the *Squadra Mobile di Catania* recorded minutes of the mortuary activities and documented clothes and personal items (ID cards, SIM cards and phone numbers) that could help the identification procedures. The Forensic Science Department of the Police (*Polizia Scientifica*) contributed to the recoding activities and photographs of the cadavers and personal effects. Over 200 documents were recovered, both associated to individuals (inside the clothes) and scattered inside the wreck: these documents suggest that people from Mali, Ivory Coast, Senegal, Eritrea, Chad, Ethiopia, Gambia, Guinea-Bissau, Somalia and Sudan were travelling on the boat (Fig. 10). Other countries of origin may reasonably be represented, but no related evidence was found. In addition, at least 700 personal belongings are associated to the articulated individuals and 400 to the disarticulated remains.

Operation Melilli consisted in the postmortem examinations of 528 bodies and body parts (named PM) onsite that were later buried and for which bone samples (mostly from the femora) were taken for DNA typing, as well as other samples for anthropology and odontology. The analysis of the commingled remains in the lab subsequently brought the number of PM individuals represented to 547, which was estimated on the number of articulated *os* coxae, since 19 additional individuals represented by hip bones and femora were reassociated from the commingled set. Additionally, the 24 bodies that were transferred to Malta in the immediate aftermath of the disaster should be included in the toll, which is now 571. Moreover, 320 crania were also found and should be taken into account- eventually the crania should be reassociated to those PM individuals that did not present the cranium. Therefore, since the reassociation of crania to PM individuals is not complete, at present the overall number of victims could amount to 891, if none of the skulls matched any of the other bodies. Nevertheless, numbers may change and will be verified by the results of the on-going DNA reassociation. So far, genetic analysis yielded 848 genetic profiles or partial profiles from the PM and the crania. The additional 19 individuals from the commingled set are still to be sampled for DNA.

Several age groups are represented by the PM individuals, from late childhood to late adulthood – the youngest individual is estimated to be approximately 10 years old, based on the unfused status of the os coxae, the oldest over 40. Sex estimation from the pelvis determined that all PM individuals were male. Morphometric analyses of the crania classified some as females [34, 35], in contrast to DNA that identified only males both in the disarticulated crania and PM individuals. Contrasting results were found for population affinity estimation as well, where metrical analyses classified most crania as African forms (Somali or Western Africa), in agreement with novel genetic analyses that described an African sample [34], whereas morphoscopic traits identified several European forms. Nevertheless, it was demonstrated that this sample is consistently different than the available reference collections [35], which represents a significant setback for the creation of reliable biological profiles.

As concerns the 30,000 commingled remains, a pilot project is focusing on the sorting and re-association of the remains recovered from the engine room, which is supposed to be the most sealed compartment of the boat. The reassignment of commingled remains to their respective PM body of origin is however extremely challenging because the bodies were buried. As a result, the pair-matching and reassociation of articulating bones may remain only partially resolved. For example, when a PM body was recovered with an incomplete vertebral column, the upper vertebra was taken to reattempt the reassociation with the commingled remains. Unfortunately, the tests performed so far allowed to reassociate only a few portions. Moreover, the extensive intermixing and the homogeneity of the sample (similar age groups and same sex) hinders the complete sorting and resolution of this commingled set.

The close partnership between LABANOF, the genetic laboratories and the ICRC and CRI deployments allowed for the identification of some victims. The main conclusion however is that so far, 33 people represented by PM individuals or disarticulated crania were identified, most of them in 2023, after the COVID and after most of the PM and crania sequencing. One must keep in mind that apart from initial grants from NGOs, no funding was ever given hence the difficulty in genetic analyses. Most likely, this number will increase as the genetic analyses are completed and compared, and more families are contacted.

## Discussion

With the increasing number of shipwrecks and countless fatalities that occur daily especially in the central Mediterranean Sea, forensic practitioners must face unique and highly demanding mass disasters related to the current migration crisis [7]. This is the case of Operation Melilli, where the set-up of a huge coordinated and multidisciplinary task force allowed for the recovery and autopsy of the victims of April 18th, 2015. By illustrating for the first time the recovery, postmortem and ongoing identification procedures, this paper underlines several fundamental issues. The large-scale operation, initiated by a government agency and involving the recruitment of various forensic specialists from Italian universities, enabled comprehensive autopsies to be performed on all recovered remains, both complete and incomplete. This effort not only ensured a thorough medico-legal investigation but created a model for a possible task force to flank UCPS in cases where prosecutors -who are not obligated to order autopsies in cases of shipwrecks- may not do so, or when only external examinations are requested as in the case of the shipwrecks in October 2013 where bodies were only examined externally and sampled for DNA [9, 10]. By institutionalizing this approach, it would be possible to ensure a more consistent and humanitarian response to future maritime tragedies. Operation Melilli thus stresses the importance to draw the public prosecutors’ attention to this urgency so that they may request postmortem examinations for each individual, in order to obtain more elements that would increase the possibility of identification. Furthermore, this mission highlights how cooperation of different institutions and professional figures can result in the successful completion of such a challenging operation. It also stresses how the lack of funding can severely hinder identification for years or forever, even when it is possible, and families are still looking for their relatives. Within the context of migration, this is the only European humanitarian operation where the wreck was recovered, governmental, non-governmental agencies and Universities pitched in and committed to implement data collection and to advance the identification procedures, providing the material and the expertise for the postmortem examinations, the genetic analyses, and the collection of AM information.

The shipwreck of April 18th, 2015, is an unprecedented event in the current migration crisis, and it can be viewed as the most tragic mass disaster that occurred in the Central Mediterranean Sea, based on the number of victims and the appalling conditions in which these people travelled. From a DVI perspective, it can be classified as an open manifest as the exact number of casualties is unknown [57], which is further complicated by the high number of bodies, the recovery site, the time of submergence and the degree of commingling. Since the main deck was an open compartment that communicated with the open sea, some remains were possibly not recovered due to marine currents. Most PM individuals and commingled remains were retrieved from the non-sequestered environments of the wreck, although the extensive decomposition, disarticulation and intermixing of the remains in the sequestered compartments represented the most challenging task for the recovery operations and for the sorting of the remains by anthropological means. In addition, for safety reasons, the recovery of the human remains could be carried out only by the Fire Department staff without a background in anatomy and osteology, who had been given only a quick one day forensic pathology briefing, and in the shortest possible time, to avoid the loss of information caused by the progressive effects of decomposition. Thus, the recovery strategy was the result of a compromise between the speed of decomposition and the safety limitations imposed by the fire brigade. Given the above-mentioned complexities, the forensic practitioners had to draw up new strategies to document the mortuary analyses and to inventory all the skeletal material, both in anatomical connection and completely disarticulated. The available DVI, ICRC and *Ricerca Scomparsi* (RiSc) postmortem forms were only partially suitable for this disaster as they do not permit the recording of several supernumerary remains that could not be associated to specific individuals. Therefore, new recording forms had to be created based on different existing forms and completed with sections for documenting disarticulated or partially disarticulated body portions, including details for the clothes and personal belongings associated to these remains.

In other mass disasters, such as the 2004 Tsunami in Thailand [58] or plane crashes involving citizens of large western countries [59, 60], the Interpol of the different countries willingly and quickly deployed the task forces for the PM and AM data collection and the Governments covered all the expenses [19]. Although the challenge here is not much different, when migrants from war-torn countries or repressive political situations are forced to risk and sometimes lose their lives crossing borders, such a setup does not exist, thus leaving the victims forsaken and disregarded [11, 12]. Few examples exist, such as October 3rd, 2013 [9] and Pylos [61], which however do not present the extensive deployment of governmental agencies (e.g., recovery of the wreck) and such complexity due to commingling and numerosity of the victims. Although they are not concluded, the operations for the 2015 shipwreck required a considerable financial commitment which was partly covered by the Italian Government (i.e., recovery of the wreck and bodies and management of the activities) and by the pro bono work of the Universities. Ten years from the disaster, and nine after recovery of the wreck, identification has reached a momentum only in the past few years when profiles have been sequenced, and families have been contacted. To date, 33 victims have been identified. Main obstacles have been funding, getting in touch with families and extensive commingling of remains.

Operation Melilli was not conceived only with the expectation of identifying all victims of the shipwreck. Rather, it functioned as a proof of feasibility, demonstrating that even in extremely complex scenarios involving deep-sea recovery, advanced decomposition and extensive commingling, it is possible to implement structured postmortem investigations, collect usable biological samples, contact families and initiate the identification processes. In this sense, the main outcome of the operation is not limited to the number of identifications achieved so far, but it lies in demonstrating that the technical barriers can be effectively overcome, and that the remaining limitations are largely related to resources, coordination and sustained long-term commitment. Given the exceptional scale, logistical complexity, and multidisciplinary coordination required for this operation, it is important to question whether similar large-scale and resource-intensive initiatives are likely to be undertaken again within the European context. Even though this may seem unlikely at this moment, one should not conclude that such identification efforts are unfeasible. Indeed, Operation Melilli explicited the need to move beyond exceptional one-off interventions, towards the establishment of stable and coordinated infrastructures for the management of migrant deaths. This includes the systematic collection and integration of postmortem and antemortem data, the development of interoperable databases, sustained funding especially but not only for genetic analyses and long-term collaboration with international organizations involved in family tracing. In this sense, the challenge is not to reproduce the scale of Melilli in every future disaster, but to translate its methodological and organizational achievements into a sustainable and continuous framework. Only through such an approach will it be possible to ensure that identification efforts become a routine component of the response to migrant fatalities, rather than an extraordinary exception.

On a final note, for the first time in the current migration crisis, Italy had the courage to face this overwhelming mass disaster and demonstrated that humanitarian rights to identity can be granted to all, regardless of country of origin [10, 12, 62–64]. To put it in the words of the Prime Minister at the time “We are human, there are 500, 600 people, children who died in this manner. I think it is right for ltaly to reveal its centuries of civilization. Let’s go and recover that boat […] I want the entire world to see”.

## Conclusions

On April 18th, 2015, a boat reportedly carrying approximately 1,000 migrants sank in the Strait of Sicily. This catastrophic event prompted one of the most extensive and complex recovery and postmortem operations in recent history. A total of 528 complete or partially complete bodies were recovered, alongside 30,000 commingled human remains. The victims were predominantly young men from Sub-Saharan Africa. A decade after the tragedy, 33 individuals have been positively identified, 40 further individuals are being confirmed through DNA, while the majority remain nameless; however identifications are still ongoing and will continue.

What sets this operation apart is that, in the broader context of the ongoing humanitarian crisis caused by migration across the Mediterranean, Operation Melilli marked the first humanitarian mission in the Mediterranean in which the Italian Government authorized the recovery of a sunken vessel with the explicit goal of identifying all the victims. Italy has so far been the only nation to recover a sunken migrant ship. This initiative represented a landmark moment in the country’s approach to migration-related deaths. Italian governmental agencies and academic institutions worked— and continue to work—tirelessly on the front lines, driven by the commitment to restore dignity and the right to identity of the deceased.

However, tackling this crisis requires more than isolated interventions- it calls for a sustained, coordinated, and multidisciplinary approach at both the national and international levels. Operation Melilli proved that Disaster Victim Identification (DVI) protocols can be effectively applied to Mediterranean shipwrecks, even though their implementation has, until now, been irregular and insufficient. Moreover, the mission highlighted the urgent need to establish and institutionalize a dedicated task force for postmortem and antemortem data collection. Such a unit would ensure a rapid, consistent, and methodologically sound response to future tragedies, aligning with international DVI standards. Operation Melilli not only set a precedent:it underscored the ethical imperative to respond to these ongoing migrant shipwrecks with humanity, scientific rigor, and resolve. The question is no longer whether identification of dead migrants is possible, but whether there is the will to implement it.

## Data Availability

Not applicable.
